# Structural characterization of SnO nanoparticles synthesized by the hydrothermal and microwave routes

**DOI:** 10.1038/s41598-020-66043-4

**Published:** 2020-06-10

**Authors:** J. S. Dias, F. R. M. Batista, R. Bacani, E. R. Triboni

**Affiliations:** Escola de Engenharia de Lorena - Universidade de São Paulo (EEL-USP), Laboratory of Nanotechnology and Process Engineering (NEP), Lorena-SP, 12602-810 Brazil

**Keywords:** Nanoparticles, Nanoparticle synthesis

## Abstract

SnO particles were synthesized by an alkali-assisted hydrothermal and microwave methods. The aqueous-based reactions were carried out at pH ~ 8, under inert atmosphere (Ar). The reactions were taken under different times, and a full XRD structural analysis was made to evaluate the conversion from the Sn_6_O_4_(OH)_4_ intermediate to SnO particles. Williamson-Hall analysis showed that the size and strain of the SnO particles were time and route treatment dependent. Microwave heating yielded a single tetragonal SnO phase after 1 h of thermal treatment, and TEM images revealed spherical-shaped SnO nanoparticles with an average size of 9(1) nm. While by the hydrothermal treatment single SnO phase was obtained only after 4 hours, yielding non-uniform and elongated particles with sub-micrometric size. A dissolution-recrystallization process was taken into account as the mechanism for SnO particles formation, in which hydroxylated complexes, Sn_2_(OH)_6_^−2^, then condense to form the oxide. The time-shorting reaction provided by the microwave-assisted synthesis may be attributed to better heat distribution.

## Introduction

Tin monoxide (SnO - Sn^+2^) and tin dioxide (SnO_2_ - Sn^+4^) are well-known semiconductors with p-type and n-type electronic properties, respectively^1–4^. Their applications have encompassed technological areas such as optoelectronics, energy storage and sensing^5–8^. SnO_2_ is a pale-yellow solid with rutile-type structure and wide-bandgap (*Eg* = *3.6* *eV*) and very used for transparent conductive electrodes^7^, gas sensors^1,8^, electrochromic devices^3^ and photoelectrodes^3,4^. While SnO is a dark solid with tetragonal structure and variable optical bandgap (*Eg* = 2.7–3.4 eV)^9–11^, and used for gas sensor devices^12^, electrodes for rechargeable Li-ion batteries^13–15^, supercapacitors^16^, native p-type conducting material^17^, as well as catalyst and photocatalyst^18,19^.

The preparation of the SnO phase is harder than SnO_2_ one, due to the favorable oxidation of the Sn^+2^ to the thermodynamically more stable Sn^+4^. Therefore, the synthetic challenge is to avoid this favorable oxidation, aside from the implementation of procedures that lead to well-controlled size and morphology. In this context, micro- and nano-crystalline SnO particles have been made by chemical/physical procedures, such as hydrothermal and solvothermal preparations, electrochemistry, ultrasound- and microwave-assisted routes, ionic liquids^19–26^, and thermal vapor deposition, thermal evaporation^3^, thermal chemical vapor deposition (CVD)^4^, and mechanical ball milling^8^. Moreover, organic additives, surfactants, inert atmosphere (Ar or N_2_) and reductive (H_2_) gases are applied with purposes of improving the yield forward the lower valence phase^15,25,27,28^.

Thermal vapor deposition and a long time of a hydrothermal treatment have been pointed out as the better conditions to obtain single-crystalline SnO particles^26,29^. Furthermore, in aqueous based-synthesis, the intermediate tin (II) oxyhydroxide, Sn_6_O_4_(OH)_4_, is often prepared from the hydroxylation of SnCl_2_⋅2H_2_O then condensation is taken on elevated temperatures and pressures, in alkaline solutions, or suspensions, to ensure due to speciation for SnO formation^30,31^.

Herein, conventional- and microwave-based hydrothermal treatments were used to form SnO nanoparticles (SnO-NPs), under inert atmosphere and relatively lower pH (pH ~ 8). The synthesis efficiency was compared in terms of reaction time, size and morphological aspects. Microwave heating yielded SnO-NPs with a spherical shape, small average size, and high uniformity upon 2 hours of thermal treatment. Conversely, hydrothermal preparation provided elongated sub-micrometric SnO-NPs with different sizes and morphologies upon 4 hours of treatment. The evaluation of the crystalline structures, from Sn_6_O_4_(OH)_4_ to SnO, in the different reaction times, was determined using X-ray diffraction microstructural analysis and transmission electronic microscopy (TEM) images.

## Experimental

### Materials

Tin chloride dihydrate (SnCl_2_.2H_2_O), ammonium hydroxide (NH_4_OH) and hydrochloric acid (HCl) were purchased from Synth, were used without further purification. The water used was double distilled and deionized.

### Synthesis of the tin (II) oxyhydroxide (Sn_6_O_4_(OH)_4_) precursor (**1**)

As a general procedure tin chloride dihydrate (0.5 mol, SnCl_2_.2H_2_O) was dissolved in 100 mL of 0.5 M HCl solution (pH ~ 2) which was then transferred to a 300 mL three-necked round bottom flask and kept under stirring at room temperature and inert atmosphere (Ar). Then, an NH_4_OH solution 28–30% (~25 mL) was added dropwise into the acidic solution until pH ~ 8, producing a white cloudy suspension (~125 mL) of the precursor Sn_6_O_4_(OH)_4_, **1**.

### Microwave treatment

**Figure 1:**
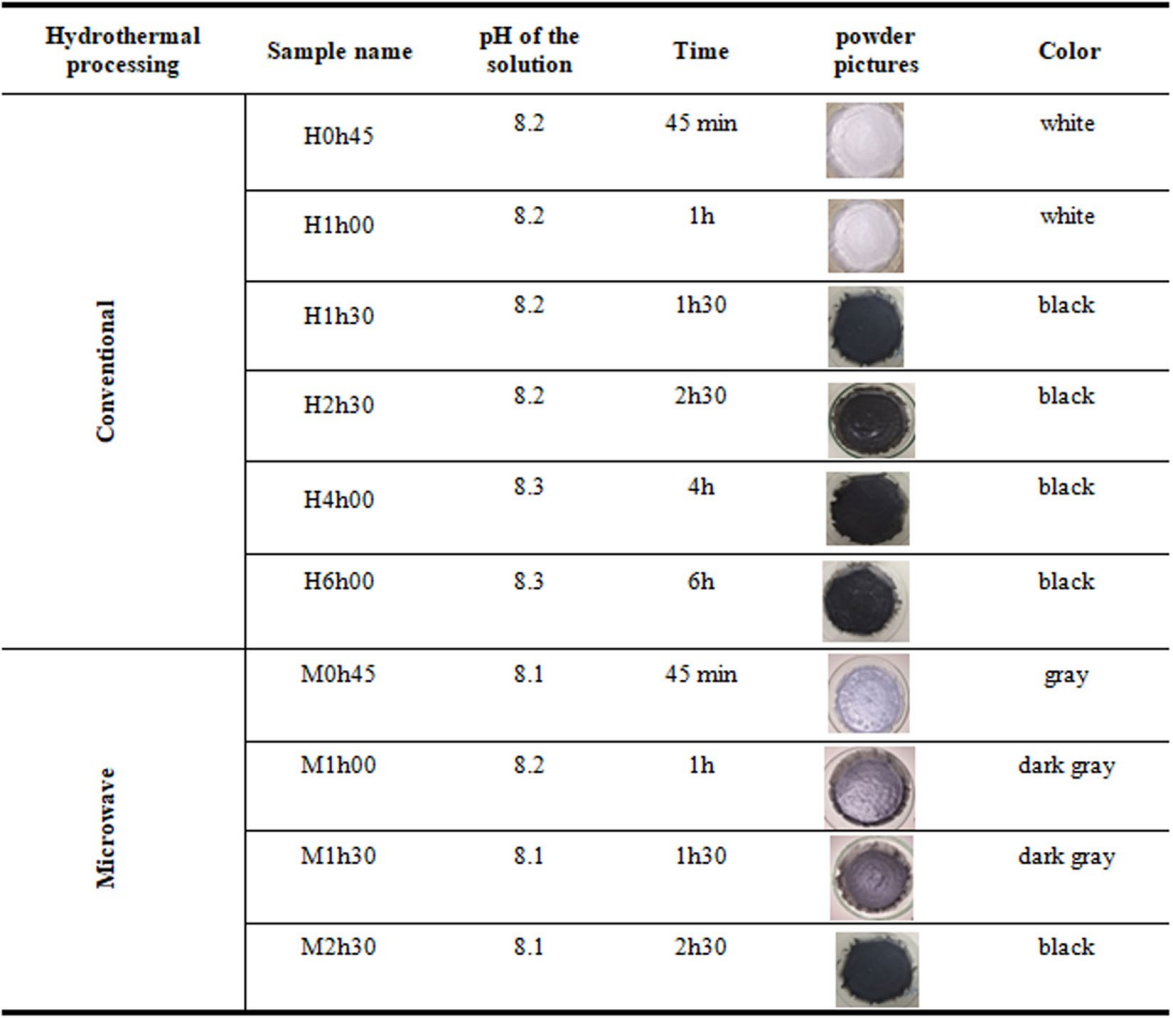
Powders obtained under various reaction times; sample names and characteristics.

A Titan MPS microwave extraction and digestion system (PerkinElmer) with 2.45 GHz magnetrons that provide up to 1,500 W of power supply. The equipment was set up with a ramp at the temperature of 80 °C, which was reached after 10 min with a maximum pressure control of 35 bar and an average of 20% output power. Reactions were carried out into Teflon autoclave reactors which were loaded with 30 mL from **1**, then heated and left for 45 min, 1 h, 1h30min, and 2h30min (sample names showed in Fig. 1). Previously, all loaded-autoclaves were purged with argon for 10 min to maintain an oxygen-free medium. At the end of each reaction, the vessels were left to chill until room temperature. The powders were filtered off and washed several times with deionized water and dried in a muffle oven in 60 °C before characterization.

### Hydrothermal treatment

For each reaction, 30 mL of the suspension **1** was put into hydrothermal autoclave reactor with Teflon chamber and also purged with argon to obtain an inert medium, then placed in an muffle oven already pre-heated at 100 °C, for 45 min, 1 h, 1h30 and 2h30, 4 h, and 6 h (sample names showed in Table 1). After cooling until room temperature, all samples were filtered off and washed several times with distilled water and dried in a muffle oven in 60 °C before characterization.

### Nanoparticles characterization

The structural characterization was performed with X-ray diffraction (XRD) measurements of the dried powder samples using a PANalytical Empyrean equipment, with a copper tube (λ = 1.5418 Å), Ni filter, and PIXcel^3D^ detector, operating at 40 kV and 30 mA, with 2θ from 10° to 90° with a 0.02° step and counting times of 3 to 5 s/step. To quantify phases and obtain structural parameters, the Rietveld’s powder structure refinement analysis was performed^32–35^ using Fullprof^36,37^ and X’Pert Highscore Plus (PANalytical)^38^ software. The peak shape was assumed as a pseudo-Voigt function with axial asymmetry, preferential direction of (00 l) planes, and the background of each pattern was fitted with a polynomial function of degree 5. Isotropic thermal vibrations were considered. Experimental parameters such as the sample’s displacement and absorption were also variables. The method takes a least-square routine to minimize the difference between the observed and simulated powder diffraction patterns with the R factors, goodness of fit (S_GoF_) and χ²^35^. The crystallite size and strain were calculated via the Williamson-Hall plot^39,40^.

Morphological characterization was performed with transmission electron microscopy (TEM) images, carried out in a JEOL JEM 2100 with LaB_6_ filament, 0.23 nm point resolution and high angular dark field detector (HAADF). Samples were suspended in ethanol and sonicated for 1 hour before measurement. Images were treated with ImageJ software^41,42^.

## Results and Discussion

### Structural and morphological characterization

The powders obtained under various reaction times for both conventional and microwave hydrothermal preparations are illustrated in Fig. 1.

**Table 1:** Structural paramenters from Rietveld analysis: lattice parameters (a/b, c)_T,H_, unit cell volume (V_T,H_), phase weight fraction (f_T,H_), where T = tin oxide (SnO), H = tin (II) oxyhydroxide (Sn_6_O_4_(OH)_4_).

Samples	a/b_T_	c_T_	V_T_	f_T_	a/b_H_	c_H_	V_H_	f_H_
	(Å)	(Å)	(Å^3^)	%	(Å)	(Å)	(Å^3^)	%
H0h45	—	—	—	—	7.9359(7)	9.112(1)	573.86(5)	100(1)
H1h00	—	—	—	—	7.9366(7)	9.112(3)	573.95(4)	100(1)
H2h00	3.8024(3)	4.8353(4)	69.909(3)	87.03(3)	7.950(3)	9.140(5)	577.62(8)	13(1)
H4h00	3.8015(7)	4.8349(9)	69.872(2)	100(2)	—	—	—	—
H6h00	3.8021(6)	4.8351(8)	69.895(2)	100(1)	—	—	—	—
M0h45	3.8012(4)	4.8331(5)	69.837(13)	53(1)	7.9399(9)	9.1225(12)	575.11(12)	47(1)
M1h00	3.8031(10)	4.8366(13)	69.951(3)	100(1)	—	—	—	—
M1h30	3.8029(18)	4.8358(21)	69.936(6)	100.0(8)	—	—	—	—
M2h30	3.8007(3)	4.8346(3)	69.838(8)	100.0(8)	—	—	—	—

Deviations presented between (), i.e., 3.5 ± 0.5 nm means 3.5(5) nm.

**Figure 2:**
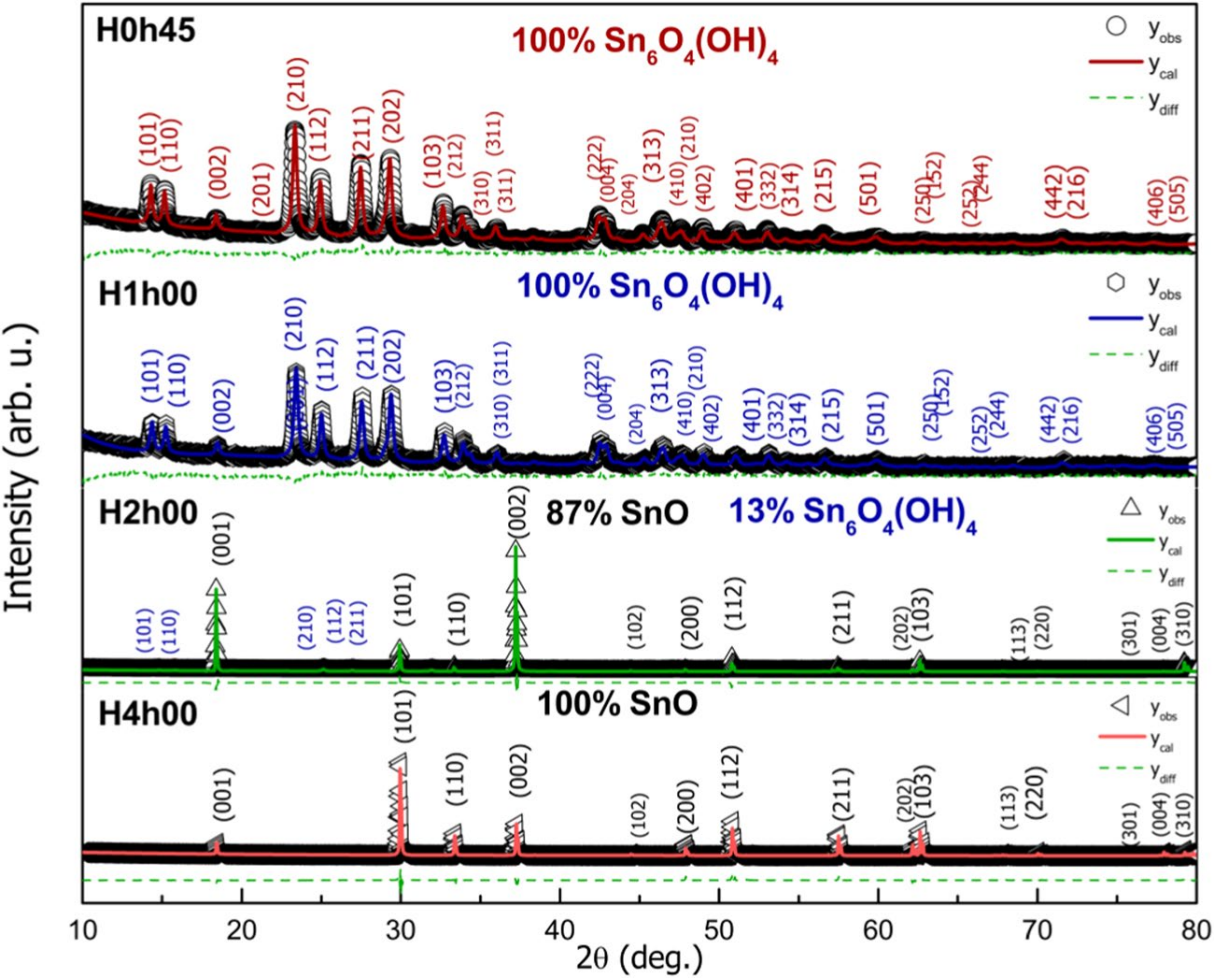
X-ray diffractograms from conventional hydrothermal (H) SnO nanoparticles. The data for H6h00 is similar for H4h00, so it was not presented in this graph. Symbols represent experimental data, the lines are for Rietveld method calculated intensity, and dashed lines denote the difference between experimental and calculated intensities.

**Figure 3:**
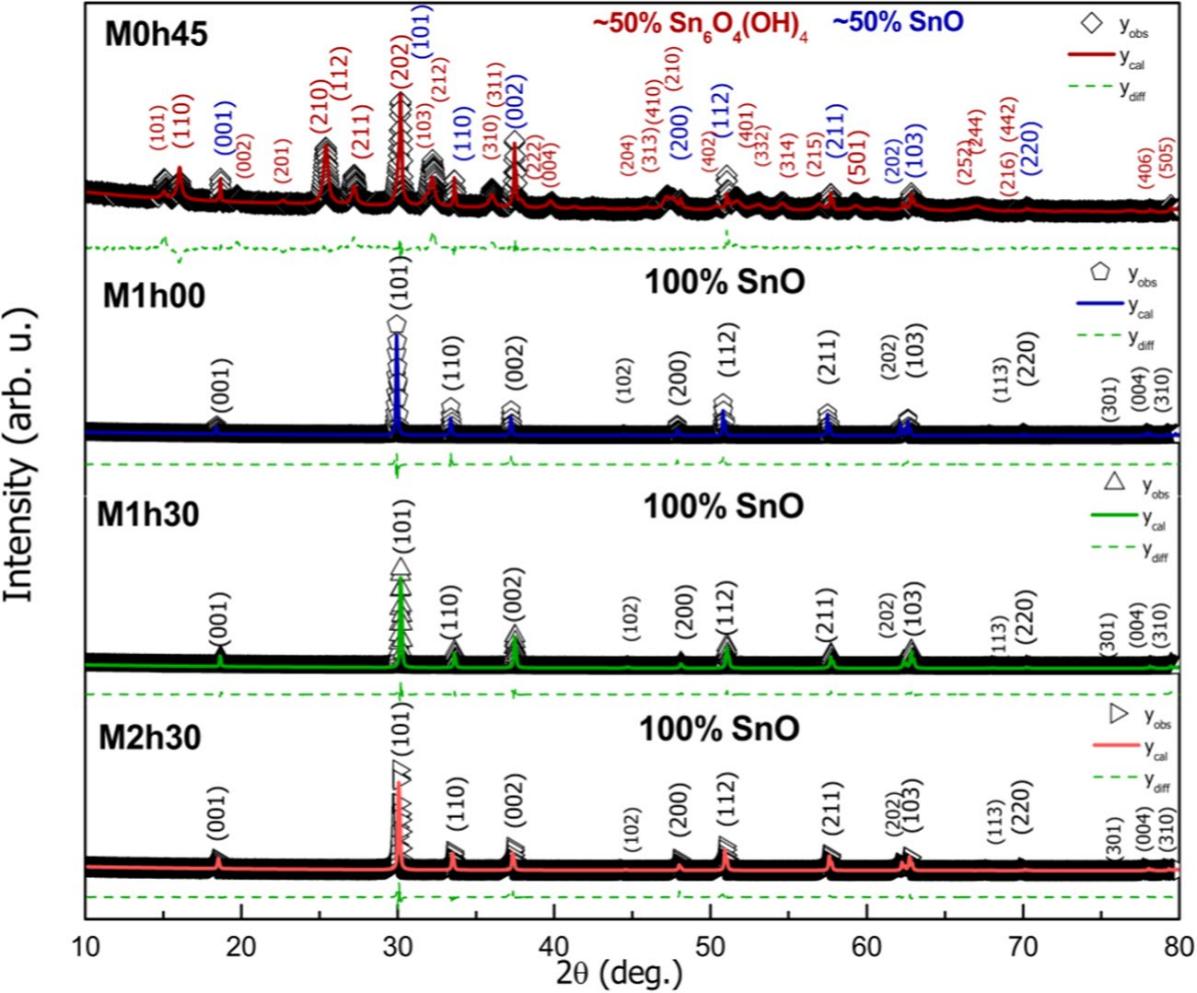
X-ray diffractograms from microwave treatment (M) SnO nanoparticles. Symbols represent experimental data, the lines are for Rietveld method calculated intensity, and dashed lines denote the difference between experimental and calculated intensities.

XRD results and Rietveld analysis are presented in Figs. 2 and 3, and Tables 1 and 2 respectively. All samples were indexed with the Inorganic Crystal Structure Database (ICSD, FIZ Karlsruhe) database^43,44^.

**Table 2:** Convergence factors of the Rietveld Method (R_WP_, R_EXP_), Goodness of Fit (S_GoF_) and global χ^2^ of the fitting.

Samples	R_WP_ (%)	R_EXP_ (%)	S_GOF_	χ^2^
H0h45	6.87	4.69	2.1	3.19
H1h00	6.78	5.07	1.8	3.21
H2h00	16.12	4.38	3.6	6.00
H4h00	7.98	5.21	2.1	4.53
H6h00	7.74	5.02	2.1	4.44
M0h45	8.47	4.94	3.0	5.20
M1h00	8.50	5.04	3.2	5.62
M1h30	7.71	5.10	2.1	4.61
M2h30	7.68	5.08	2.3	5.14

Tables 1 and 2 present the structural parameters and fraction of the phases, besides the factors of agreement of the adjustment. Since some samples presented more than one crystallographic phase, preferential high direction, and asymmetry of peaks, especially for samples synthesized via conventional hydrothermal thermal treatment, the convergence values such as goodness of fit (S_GoF_) and global χ^2^, are relatively higher than expected in general.

Quantification results show that for the conventional hydrothermal method, the complete formation from the tin (II) oxyhydroxide phase, Sn_6_O_4_(OH)_4_ (P$\overline{4}$2_1_c, PDF #01-084-2157)^43^ to pure tetragonal phase SnO (P4/nmmS, PDF #01-085-0423)^44^ happens with at least 4 hours of thermal treatment. Within 2 hours of treatment, it can be observed a large intensity change for (001) and (002) planes, suggesting the formation of rods with growth in the *c* parameter direction. Lattice parameter and cell volume are similar to those to the standard crystallographic SnO data and a slight increase with treatment time from 4 to 6 hours (Table 1). For the microwave method, there is already ~50% formation of SnO and ~50% of Sn_6_O_4_(OH)_4_ phases with 45 min of treatment. The complete formation of crystalline single-phase SnO occurs after a 1-hour thermal treatment.

**Figure 4:**
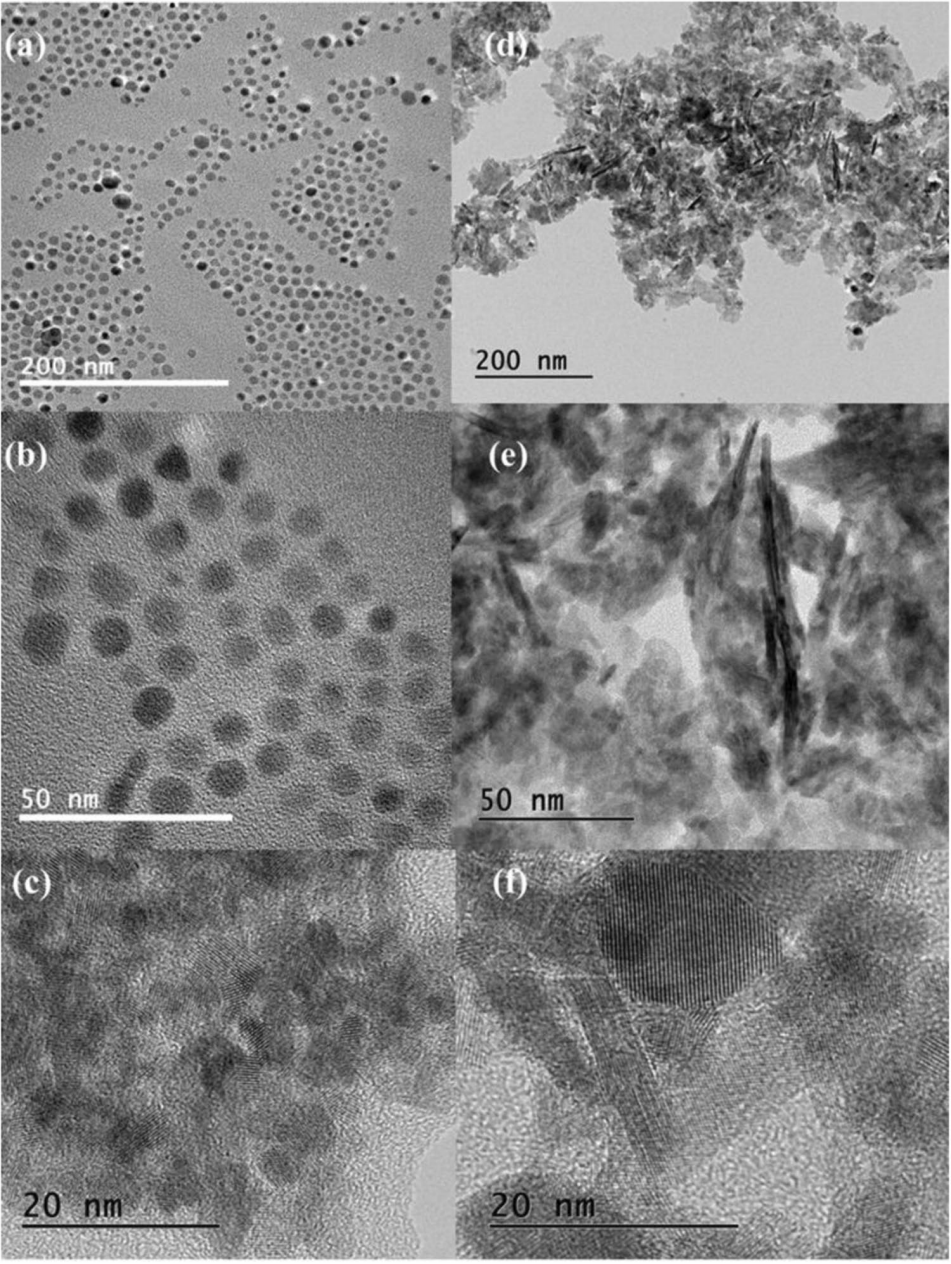
TEM images for SnO nanoparticles. (**a**) M2h00; (**b,c**) M1h00; and (**d–f**) H6h00.

Figure 3 shows TEM images from SnO nanoparticles synthesized by both methods. It can be seen that microwave-assisted synthesis leads to uniformly sized spheres, Fig. 4(a–c), while in the conventional hydrothermal treatment there are agglomerated particles without definite shape, Fig. 4(d–f). The conventional hydrothermal synthesis also produces elongated particles that could be responsible for the large intensity of (00 l) peaks. The average nanoparticle size for M1h00 is 9(1) nm and, for M2h00 is 4.0(7) nm. For the H6h00 sample, the sizes vary from 10 to larger than 50 nm, the elongated particles have an average length of 30(6) nm. However, the microwave treatment leads to a more uniform formation of SnO nanoparticles instead of the conventional heating method.

**Figure 5:**
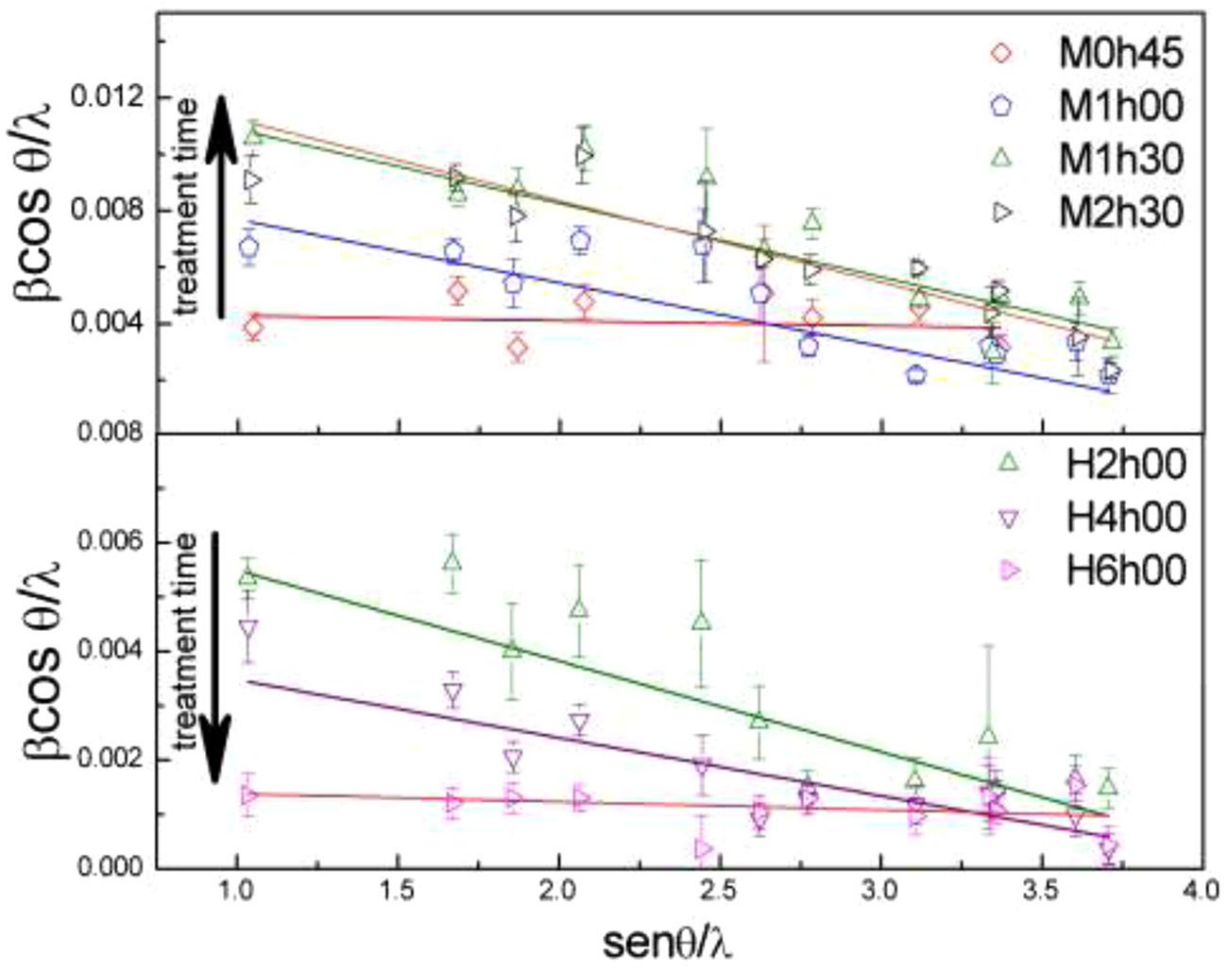
Williansom-Hall plot for SnO nanoparticles, where β stands for FWHM (full width half maximum) from the XRD peaks after instrumental correction, θ is the diffraction angle and, λ is the X-ray wavelength. (**a**) Conventional hydrothermal and, (**b**) microwave thermal treatment.

Microstructural analysis was performed in order to evaluate the average characteristics of the SnO powders. The Williamson-Hall plot (WHP) is shown in Fig. 5, and the results are displayed in Table 3. This analysis takes into account the crystallite size and the lattice deformations instead of the Scherrer formula that only evaluates the crystallite size^40,41,45,46^.

**Figure 6:**
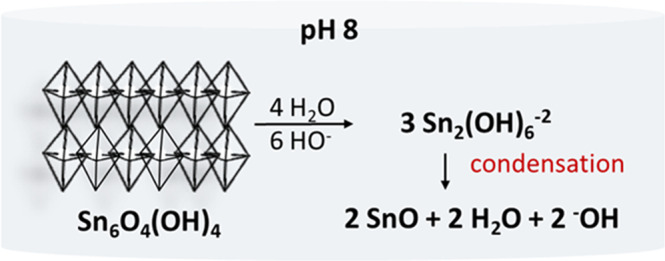
Dissolution-recrystallization steps for the formation of SnO particles from Sn_6_O_4_(OH)_4_ intermediate.

**Table 3:** Crystallite size (D_WH_) and strain (ε) determined via Williamson-Hall Method for the SnO crystal phase.

Samples	D_WH_ (nm)	ε (%)
H0h45	—	—
H1h00	—	—
H2h00	112(16)	−0.0017(3)
H4h00	176(26)	−0.0011(3)
H6h00	523(53)	−0.00015(4)
M0h45	179(19)	~0
M1h00	81(11)	−0.0023(4)
M1h30	59(4)	−0.0026(3)
M2h30	57(6)	−0.0029(4)

The crystallite size calculated is greater than TEM images mostly because of the agglomerated particles between each other (Table 1), producing an effect on the intensity distribution, since crystal sizes smaller than 100 nm are harder to analyze via XRD size-strain analysis^40,45,46^. These particle clusters can be seen in Fig. 3(c–f). The crystallite size grows with the conventional hydrothermal treatment time, while in the microwave treatment the crystallite and nanoparticle size decreases.

The linear behavior of data in the WHP is consistent with and regular distribution of crystallite sizes. In this case, for the conventional hydrothermal treatment, the negative strain can be related to particle inhomogeneities, since the strain decreases as de particle size increases and the lattice parameters increase (Table 2). For the microwave treatment, the negative strain is related to the shrinkage of the unit cell showed by the Rietveld results (Table 2), which can also be related to the decrease in the nanoparticle size^46^.

### Formation of SnO-NPs

Dissolution-recrystallization and oriented-attachment (OA) have been assigned to the formation of SnO particles with different textures and sizes^47^. In the present case, OA is ruled out because there is no evidence of an assembled architecture. Thus, SnO particles should be build up through dissolution-recrystallization way. In this pathway, the formation of SnO crystals is derived from the decomposition of the oxo-hydroxo intermediate, Sn_6_O_4_(OH)_4_, and then further condensation of the tin-hydroxo complexes showed in Fig. 5, proposed for aqueous-based preparations carried out at pH ~ 8.

Firstly, Sn_6_O_4_(OH)_4_ is dissolved by the attack of the hydroxide anion, ^−^OH, and water molecules forming the anionic Sn_2_(OH)_6_^−2^ complex, where two Sn^+2^ and hydroxo groups are bound by bridging, then it condenses to form SnO particles delivering water and hydroxide anion^47^. In this mechanism, in the overall synthesis, ^−^OH is consumed.

The synthesis efficiency may also be correlated to the favorable equilibrium in aqueous solution at pH ~8. SnO’s phase diagram, relating concentration as a function of pH, shows that solubility of the ionic oxo, HSnO^−2^, and hydroxo, Sn(OH)_2_, complexes are low (<10^−6^ M)^30,31^, thus, the equilibrium is promoted to SnO solid phase. Moreover, species of higher oxidation state can be suppressed, inasmuch as the reaction is taken under an inert atmosphere. Therefore this condition seems to be optimized to grow SnO particles in spite of the consumption of base throughout the stepwise mechanism.

### Microwave versus Conventional oven heating

The heat-generation from microwave irradiation has been attributed to two main physical processes, namely as dipolar polarization and ionic conduction. Both are accountable for the gains such as decreased reaction rate and selectivity to obtain metal oxides^48–50^. Their magnitudes depend on the bulk dielectric permittivity (ε), a measure of the extent to which a material can be polarised by an external electric field, that can be expressed as *ε* = *ε*’ + *ε*”j, where *ε*’ is the dielectric constant of the material, *ε*” is the dielectric loss and j is the imaginary number where j^2^ = −1^21^; the latter term *ε*” is related to the gain heating. Laybourn and co-workers have considered the role of the *ε* on the synthesis of MOFs under microwave irradiation and elucidate the influence of the concentration, ionic strength as salts are used, temperature, and microwave cavity as well. All these factors have been showed as significant, and even anion- and cation-ligand bond or interaction may exert a fair interference on the heating and reactivity^51^. Herein, microwave heating speeds up the formation of SnO-NPs even at a lower temperature than that for hydrothermal preparation leading to small and spherical-shaped SnO-NPs. Conversely, long particles and sheets morphologies were obtained by the conventional-hydrothermal preparation, in which heating is distributed by convection currents. Hence, one may argue that microwave delivers uniform heat through the reaction medium, which accelerates the rate of nucleation leading to small nanoparticles. On the other hand, the inhomogeneous heat spreading, under hydrothermal reaction, leads to particles of different shapes and sizes and certainly favors the ripening processes.

**Figure 7:**
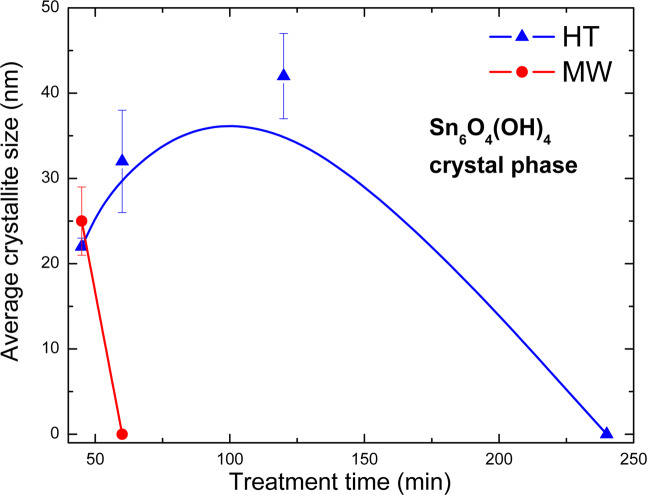
The development of the average crystallite size of the Sn_6_O_4_(OH)_4_ phase during hydrothermal and microwave treatment time.

XRD results (see Figs. 2 and 3) showed that different intervals exist for starting the conversion from Sn_6_O_4_(OH)_4_ to SnO. Under microwave, it seems to be taking place at earlier reaction time while it happens only after 1 h through the hydrothermal treatment. This delay-effect has been noticed in another study concerning reaction time dependence to form SnO crystals^49^. Three possible explanations are *i*. a previous formation of an unknown tin-based structure, after then the condensation happens; *ii*. this interval is needed to increase the concentration of the Sn_2_(OH)_6_^−2^. *iii*. a unique effect might occur under microwave irradiation. The former may be discarded since that XRD analyses only identified the Sn_6_O_4_(OH)_4_ and/or SnO phases. Likewise, the second option was dismissed toward the balance of the end up mass obtained that showed practically equal for all reaction: m = 3.50(3) g. If there was a time interval for concentrating the Sn_2_(OH)_6_^−2^ in solution, the final mass of solid would decrease in those periods before converting to SnO. Therefore, microwave irradiation should induce a specific effect on reactivity. In order to understand the development of the Sn_6_O_4_(OH)_4_ crystalline precursor phase, the average crystallite size was measured from the most intense XRD peaks for both microwave (MW) and hydrothermal (HT) synthesis, Fig. 7.

**Figure 8:**
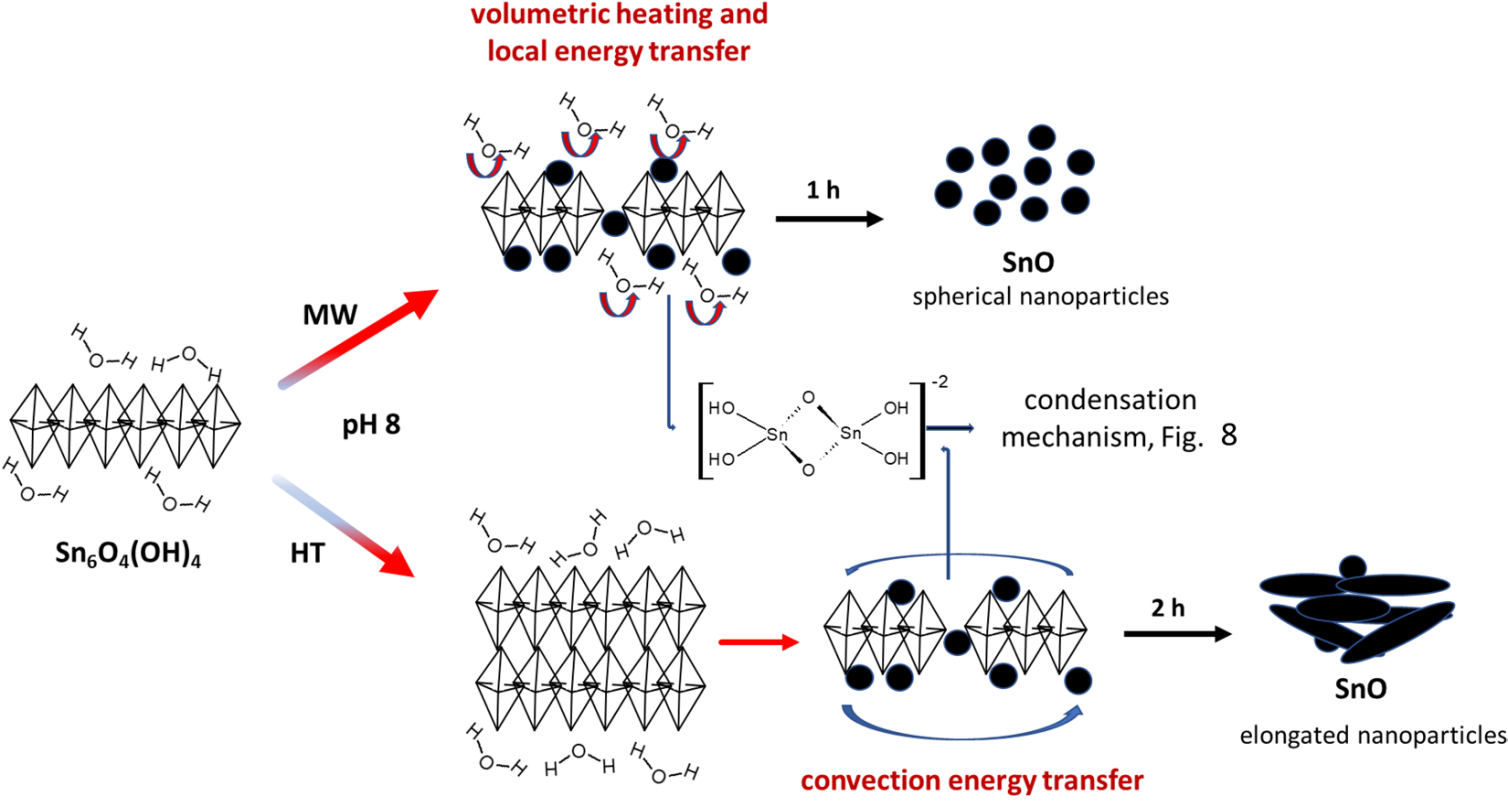
The effect of the conventional and microwave heating in the formation mechanism of SnO-NPs from Sn_6_O_4_(OH)_4_^53^.

It can be seen that a second-order event in which HT treatment induces growth of the Sn_6_O_4_(OH)_4_ crystallite size until ~80 min, then the size decreases accompanying the conversion of Sn_6_O_4_(OH)_4_ to SnO. On the other hand, in the MW treatment, there is a very quick and straight decrease along with the conversion to SnO. In addition, the WHP analysis (see Fig. 5) showed that HT crystallite tensions decrease just after 2 h whereas ones from MW crystallite get an early and straight increase. Therefore, assuming a recrystallization-dissolution mechanism in which such an Sn_2_(OH)_6_^−2^ anionic complex is formed, these findings allow to set down the following panorama, shown in Fig. 8: *i*. MW treatment speeds up the formation of the anionic complex up to its saturation, then, the condensation happens inside a uniform heat distribution, favoring faster nucleation and smaller particles; *ii*. as the HT treatment needs first to achieve an average work temperature, the precursor Sn_6_O_4_(OH)_4_ grows up in disordered directions until ~1 h, then followed by SnO particle formation. After two hours, the whole system gathers enough energy to develop the condensation step form SnO from Sn_2_(OH)_6_^−2^, in spite of the inhomogeneous heat flux.

**Figure 9:**
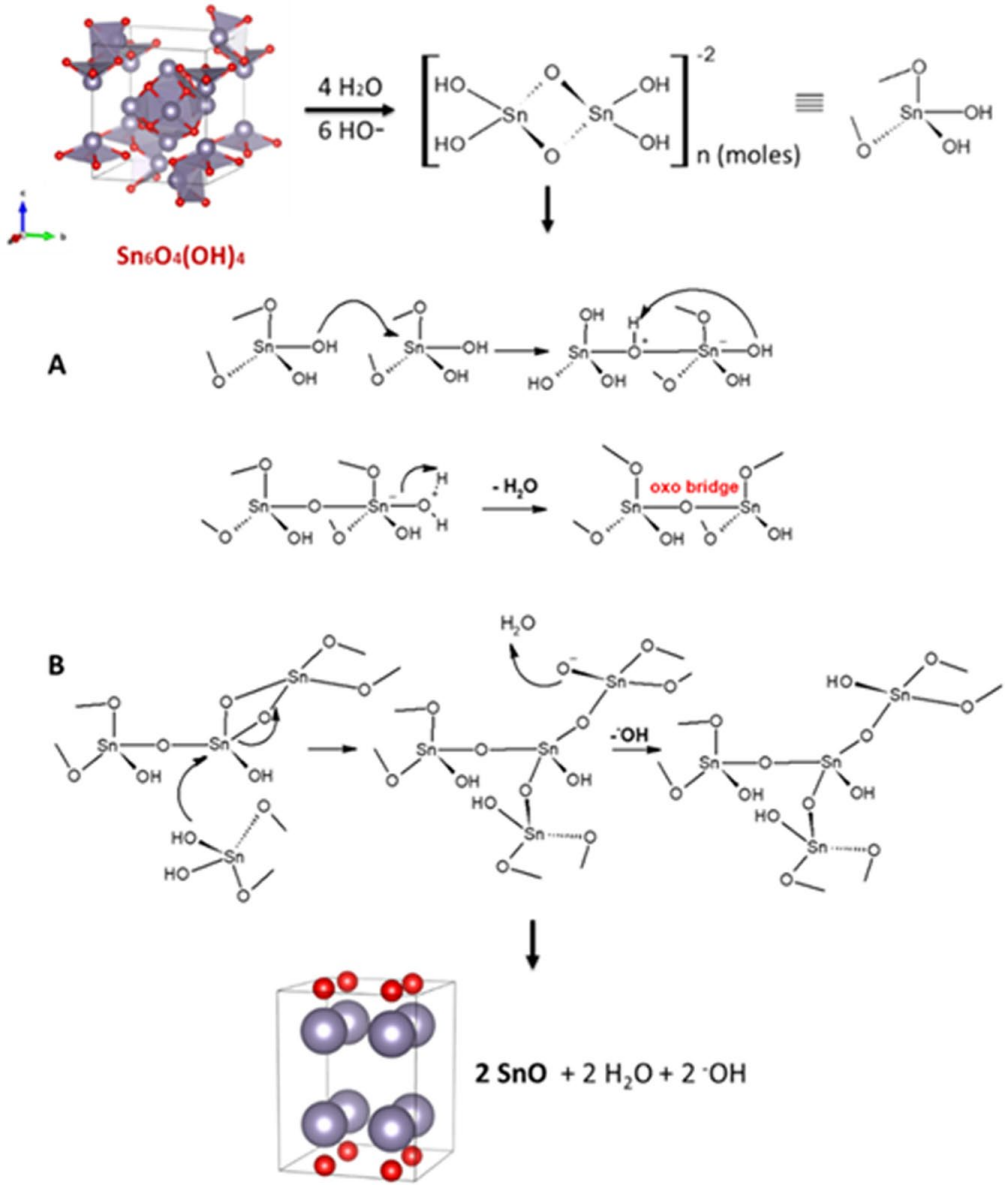
Mechanism proposition of condensation steps: the possible pathways of the dissolution of Sn_6_O_4_(OH)_4_ and oxolation of the Sn_2_(OH)_6_^−2^ to form SnO-NPs^43,44,49–54^.

Moreover, taking into account the interference that microwave assisted-synthesis can have due to the variations of the bulk dielectric permittivity (*e*)^50,51^, it is not easy to predict its action on the mechanism of condensation, but should be mainly on the steps in which water acts as attacking or living group, *i.e*., dissolution of Sn_6_O_4_(OH)_4_ and oxolation of the Sn_2_(OH)_6_^−2^. The oxolation happens through an addition-elimination with a proton-transfer step-mechanism that releases water as leaving group forming an oxo bond, Fig. 9. As seen in the literature previously, the enhanced maturation of hydroxyapatite under microwave irradiation has been driven by the strong excitation or stretching of the calcium-water bond. Furthermore, the proposed mechanism also provides a path to the final hydroxide, ^−^OH, formation^52^. In our case such an effect should take place on the stepwise in which water is withdrawn, accelerating SnO formation The tetrahedra complex Sn_2_(OH)_6_^−2^ has two oxo bridges that are attacked by the hydroxo nucleophilic group via a nucleophilic substitution, further opening a cycle, producing the tin-alkoxy, Sn-O^−^, that takes off a proton from water delivering ^−^OH.

## Conclusion

Conventional- and microwave-hydrothermal aqueous syntheses to form SnO were carried out at pH 8 under an inert atmosphere. The efficiency of conversion from Sn_6_O_4_(OH)_4_ to SnO was verified in the different reaction times by XRD analysis, as well as the structural and morphological  aspects of the particles. Conventional hydrothermal treatment yielded SnO sub-micrometric particles with sizes varying from 30 to 550 nm after 4 hours. By the microwave route, uniformly and spherical-shaped ZnO-NPs with 4 nm average size were obtained after 2 hours. XRD analyses indicate that size and morphology have a treatment time dependence. The XRD results were different from those observed by TEM, which can be attributed to samples’ preparation, leading to larger agglomerates. The mechanism of the formation of SnO may be assigned to a dissolution-recrystallization process, and the acceleration provided by the microwave-assisted preparation may be attributed to a better heat distribution into the synthesis medium.
